# Supramolecular Allostery Achieves Tunable Luminescence Aromatic Bridged Phenothiazine Noncovalent Frameworks

**DOI:** 10.1002/advs.77843

**Published:** 2026-09-16

**Authors:** Xinping Lu, Jianchong Chen, Jie Yu

**Affiliations:** ^1^ Yunnan Key Laboratory of Modern Separation Analysis and Substance Transformation College of Chemistry and Chemical Engineering Yunnan Normal University Kunming People's Republic of China

**Keywords:** cucurbit[8]uril, supramolecular assembly, supramolecular organic framework, tunable luminescence

## Abstract

Here, we report an engineering tunable luminescent supramolecular organic framework (SOF) enabled by multiple allosteric effects, composed of a tetracationic vinylphenylpyridinium‐modified aromatic‐bridged phenothiazine guest and cucurbit[8]uril in a 1:2 stoichiometric ratio, formed via non‐covalent polymerization driven by strong host‐guest cooperation. The nanofiber‐like guest is activated to form a supramolecular network polymer with negligible luminescence at 815 nm, showing an ultra‐large Stokes shift of up to 240 nm. Interestingly, under oxidant driving, the guest allosterically converts to phenothiazine sulfoxide, which makes the weakly luminescent SOF exhibit strong yellow fluorescence, and the emission blueshifts to 610 nm, with the quantum yield increasing 46‐fold to 22.2%. Additionally, the dynamic reversibility of macrocyclic bonding endows the SOFs with reversible and dissociative allosteric properties, enabling thermos‐reversible regulation of their luminescence and competitive guest‐induced quenching performance. Finally, the allosterically controlled luminescent SOF has been successfully applied to information encryption and logic gates, providing a new perspective for developing intelligent supramolecular materials.

## Graphical abstract

Tunable luminescent supramolecular organic framework enabled by multiple allosteric effects, composed of a tetracation vinylphenylpyridinium‐modified aromatic‐bridged phenothiazine guest and cucurbit[8]uril, not only exhibits unique guest oxidation‐driven NIR luminescence blue‐shift and effectively enhances the quantum yield by 46‐fold to 22.2% but also achieves the thermos‐reversible and host‐guest dissociation allosterically controlled luminescence, which was successfully applied to information encryption and logic gates.

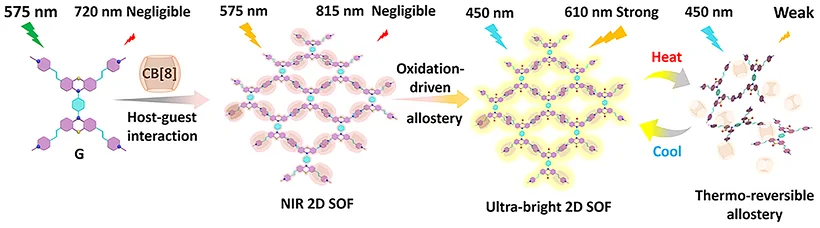

## 1 Introduction

Organic frameworks are porous materials that expand organic structural units into periodic network structures via chemical bonds or non‐covalent interactions, including metal‐organic frameworks (MOFs) [1], covalent organic frameworks (COFs) [2], and supramolecular organic frameworks (SOFs) [3]. These materials have attracted extensive attention and have been widely applied in catalysis, adsorption, separation, and luminescent materials [4, 5, 6, 7, 8]. Among them, SOFs based on macrocyclic compounds have unique advantages for the construction of dynamic, responsive materials because they can effectively encapsulate guests with symmetrical structures via various noncovalent interactions, such as hydrogen bonding, hydrophobic interactions, and electrostatics, and then assemble into periodic supramolecular structures in solution [9, 10]. Among many macrocyclic hosts, pumpkin‐like cucurbit[8]uril (CB[8]), formed by the condensation of glycoluril and paraformaldehyde, has a rigid molecular structure and large hydrophobic cavity [11, 12, 13, 14], which can encapsulate two cation aromatic molecules in a head‐to‐tail mode and act as a cross‐linking reagent for supramolecular polymers. Many functional SOFs have been developed based on these features of CB[8]. For example, Li group reported a series of CB[8]‐mediated multidimensional SOFs [15, 16]. They synthesised phenylpyridinium‐modified tetraphenylmethane as a cationic guest, which can be effectively encapsulated by CB[8], and further assembled to form 3D polycationic SOFs with a multidimensional pore structure, showing selective absorbability for drugs and dyes, then applied to the photodynamic agent cargo for controlled releasing and served as an antidote for heparin [17, 18]. Cao et al. employed tetra‐arm cationic tetraphenylethene or hexaphenylbenzene as the monomer cross‐linker with CB[8] to form fluorescence‐enhanced SOFs, then cascade‐assembly with peptides as chiral sources to construct a chiral transfer supramolecular system [19, 20]. Ma reported that bromophenylpyridium‐modified tetraphenylmethane could be encapsulated by CB[8] in a 1:2 stoichiometric ratio and assembled to form a 3D phosphorescent SOF that served as a donor to load dyes, forming a phosphorescent resonance energy transfer system [21]. Li et al. synthesized a tricationic radical derivative as the guest and assembled it with CB[8] to form a stable near‐infrared luminescent SOF in aqueous with a quantum yield of 5.0% [22]. Thus, the host‐guest interaction of CB[8] can not only construct an ordered supramolecular structure in solution but also confine the guests to promote the luminescence of the assembly, forming excellent luminescence‐performance SOFs. Despite several reports on fluorescence or phosphorescence SOFs, the assembly of responsive guests as allosteric factors with macrocyclic hosts to form noncovalent framework materials featuring supramolecular allostery‐controlled luminescence properties is still rarely reported.

**SCHEME 1:**
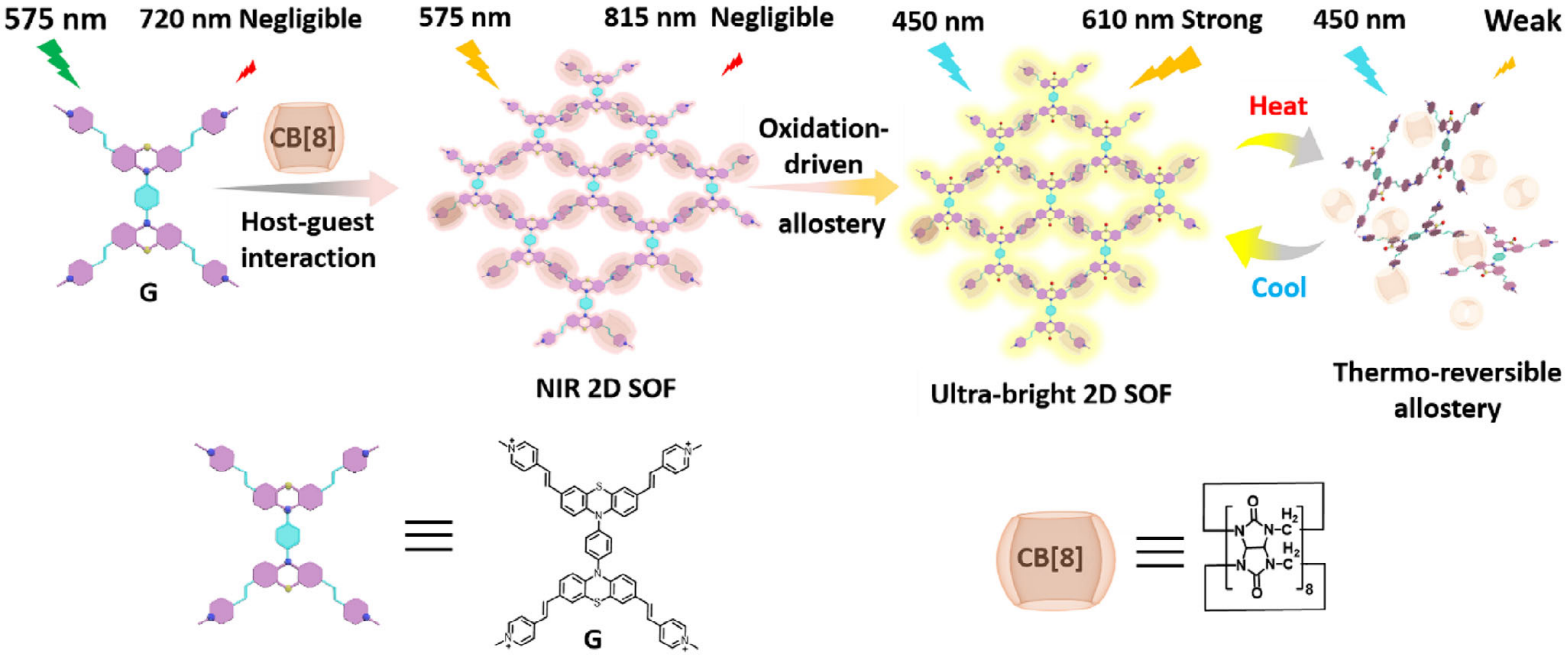
Supramolecular allostery achieves tunable luminescence aromatic bridged phenothiazine noncovalent frameworks.

In this work, the covalent linkage of the electron‐withdrawing pyridinium group and the electron‐donating phenothiazine unit yields a conjugated extended vinylpyridinium modified phenyl‐bridged phenothiazine, which serving as both a monomer and an allosteric factor for the luminescent SOFs (Scheme 1). Taking advantage of the large cavity of CB[8] that can effectively bind two mono‐cation guests, the cationic arms of the tetra‐cation phenyl‐bridged phenothiazine are encapsulated by CB[8] to form a *J*‐type aggregated supramolecular linker, and further undergo non‐covalent polymerization to form a periodic supramolecular structure with excellent luminescent properties. Notably, oxidation of the phenothiazine sulfur to sulfoxide imparts oxidative allostery‐modulated luminescence to the SOF. At the same time, the dynamic reversibility of macrocyclic assemblies allows for convenient investigation of both thermal‐reversible and host‐guest allosteric regulation of SOF luminescence. Therefore, the allosterically tunable phenylphenothiazine SOF, leveraging its stimuli‐responsive luminescence, holds promise for applications in information encryption and complex logic gate systems.

## 2 Results and Discussion

**FIGURE 1:**
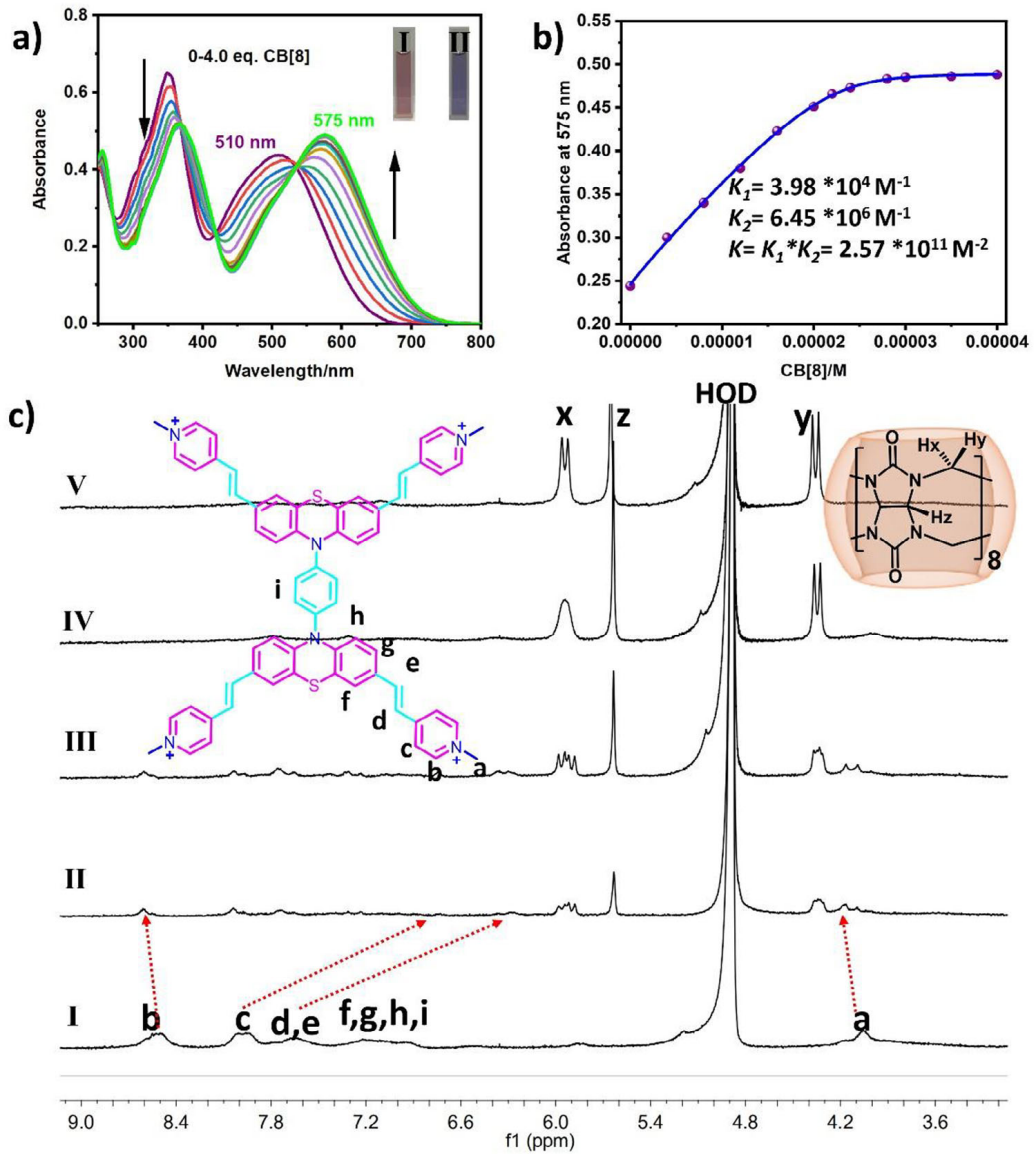
(a) UV–vis spectra of G in the presence of different mount of CB[8] ([G] = 10 µm), inset: photographs of I) G and II) G/CB[8] solution. (b) Nonlinear least‐squares analysis of the absorbance intensity changes of G at 575 nm with the addition of CB[8] to calculate the *K* value between CB[8] and G. (c) ^1^H NMR septra of G with the addition of CB[8] ([G] = 0.2 mM, I): 0 eq.; II):0.5 eq.; III):1.0 eq.; IV): 1.5 eq. V): 2.0 eq. CB[8]) in D_2_O/(CD_3_)_2_SO = 95/5.

First, the tetrabromophenyl phenothiazine was reacted with 4‐vinylpyridine to give tetra‐vinylpyridine modified phenothiazine by coupling reaction under palladium catalysis (Scheme S1). Then, undergo a nucleophilic substitution reaction with methyl iodide and exchange the anion to give tetracationic vinylphenylpyridinium phenyl‐bridged phenothiazine as the guest (G). The chemical structure of G was characterized by nuclear magnetic resonance spectroscopy (NMR) and high‐resolution mass spectrometry (Figures S1–S5). Next, the host‐guest properties of G/CB[8] were investigated by UV–vis spectroscopy and NMR experiments. With the addition of CB[8], the characteristic absorption peak of G at 510 nm gradually decreased, while the absorption peak at 575 nm increased, resulting in a 65 nm red‐shift (Figure 1a). The solution of G changed from light red to deep purple in the presence of CB[8] (Figure 1a, inset), suggesting the formation of charge‐transfer complexes [23]. The inflection point of the Job curve of G/CB[8] is located at 0.33 (Figure S6), indicating CB[8] binds with G at a 1:2 stoichiometric ratio [24], ascribing to CB[8] possesses a large hydrophobic cavity that can encapsulate two mono‐cation aromatic molecules [25]; thus, the guest containing four cations can be encapsulated by CB[8] at a stoichiometric ratio of 1:2. According to the change of the absorption peak intensity of G at 575 nm (Figure 1b), the binding constant of G/CB[8] was calculated to be 2.57 × 10^11^ M^−2^ [26, 27], which is convenient for G/CB[8] further non‐covalent polymerization to form the ordered assembly.

Furthermore, the binding mode between G and CB[8] was studied by NMR experiments. In Figure 1c, the free G gives broad proton signals, attributed to the self‐aggregation of tetracationic vinylphenylpyridinium phenyl‐bridged phenothiazine in solution. In the presence of CB[8], the protons H_c_ and H_d‐e_ belonging to the vinylpyridinium of the guest shift from 7.97 and 7.65 ppm to 6.73 and 6.29 ppm, respectively, manifesting that the vinylpyridinium is located in the CB[8] cavity‐the shielding region [28]. However, the proton H_b_ on the cationic side arm and the proton H_a_ belong to the methyl group give a downfield shift of 0.10 and 0.11 ppm, indicating that the vinylpyridinium and methyl are located at the axial port of CB[8] [29]. As the ratio of CB[8] increases to 2.0 eq., the proton signal in the guest becomes broad, ascribing to G/CB[8] assembly to form large aggregates [30]. In addition, the 2D NOESY spectra show that the related signals between the protons in the aromatic region of the guest and CB[8] were found at 6.75–7.75 ppm and 4.0–4.50 ppm (Figure S7), confirming that the cationic arms of G were included by CB[8].

**FIGURE 2:**
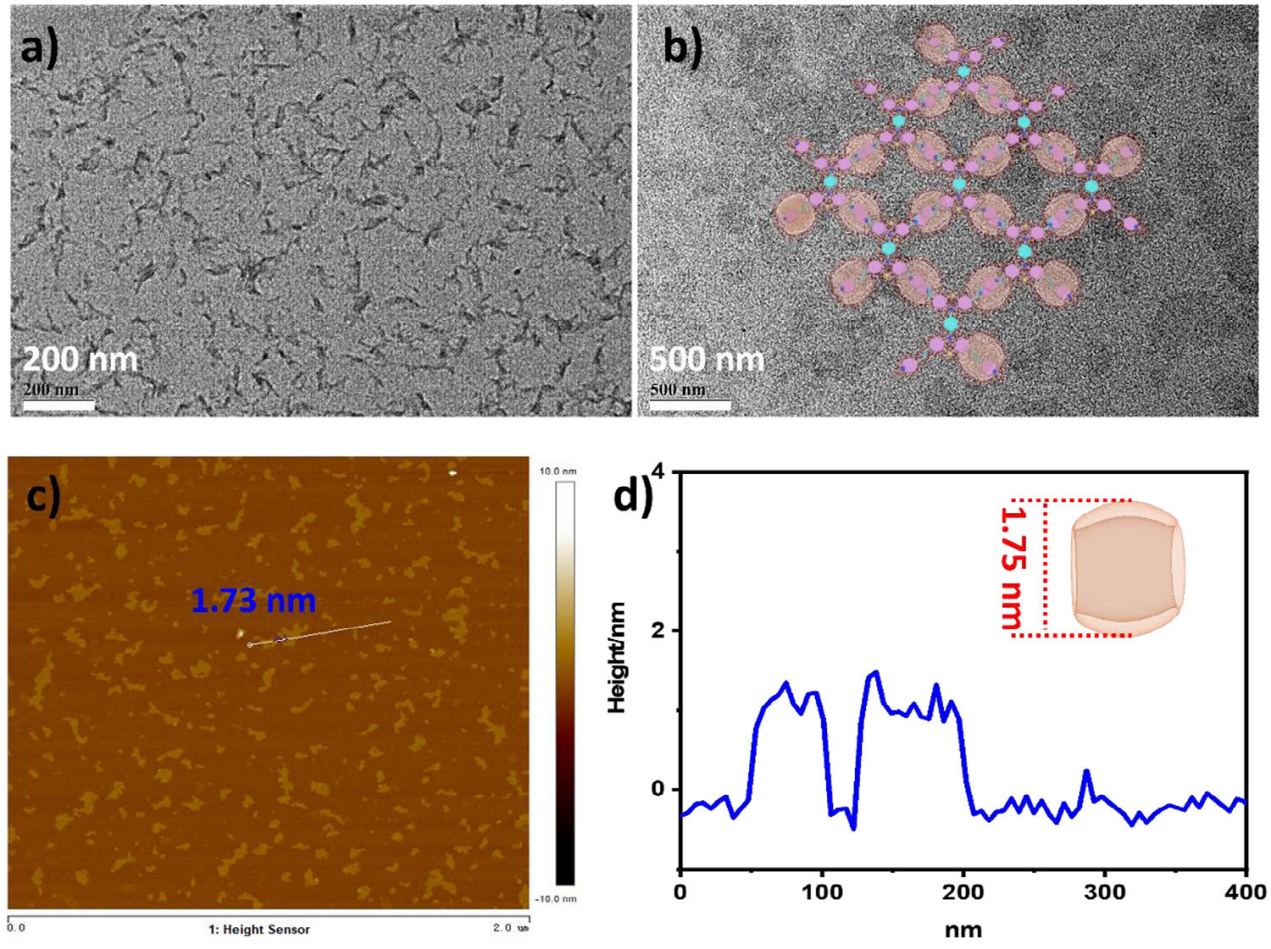
TEM images of (a) G and (b) G/CB[8], inset: schematic illustration of the assembled model of G/CB[8]. (c) AFM images of G/CB[8]. (d) The height section profile of G/CB[8], inset: theoretical outer diameter of CB[8].

Transmission electron microscopy (TEM), atomic force microscopy (AFM), and dynamic light scattering (DLS) were performed to investigate the assembly behavior of G/CB[8]. The nanofiber morphology was observed in the TEM image of G (Figure 2a), ascribing to the free tetracationic guest with a hydrophobic phenyl‐bridged phenothiazine core, which can self‐assemble into aggregates. In Figure 2b, G/CB[8] gives nanosheet‐like morphology with sizes ranging from 100 to 600 nm. The thin nanosheet was observed in the AFM image of G/CB[8] (Figure 2c), and the thickness of the nanosheet structure was tested as 1.73 nm (Figure 2d), close to the outer diameter of CB[8] (1.75 nm) [31, 32], indicating that the host‐guest interaction of CB[8] drives the guest from a fibrous morphology to a single‐layer network nanostructure. In the zeta potential test (Figure S8), the guest G gave +17.0 mV, and the assembly of G/CB[8] was +13.8 mV. Compared with G, the hydrodynamic size of G/CB[8] increased to 513 nm (Figure S9), further confirming the tetra‐cationic guest transfers to the larger assembly upon the activation of CB[8].

**FIGURE 3:**
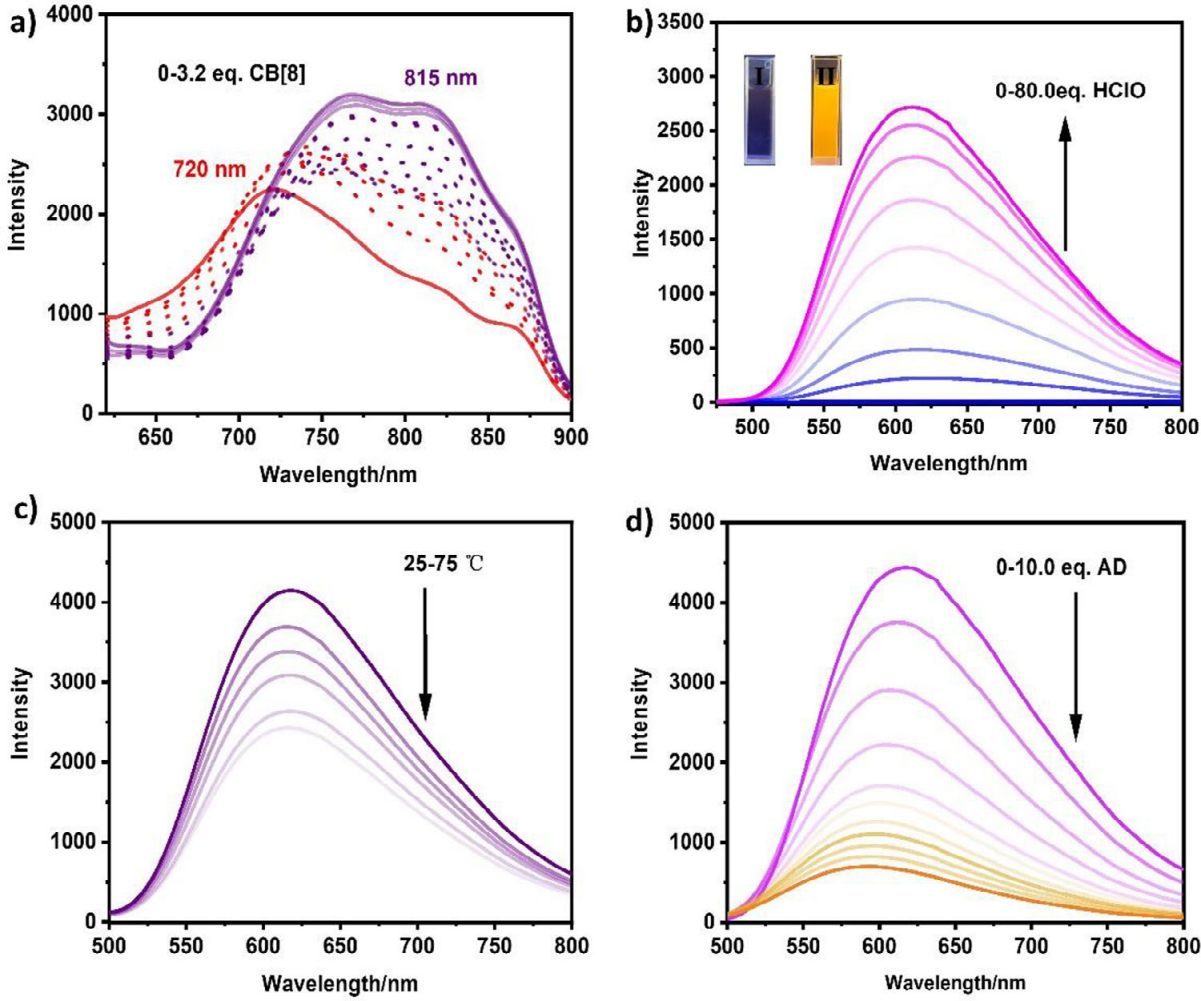
(a) Fluorescence spectra of G in the presence of different mount of CB[8] (λ_ex_ = 575 nm, 0–3.2 eq. CB[8]). (b) Fluorescence spectra of G/CB[8] in the presence of different mount of HClO (λ_ex_ = 450 nm, 0–80 eq. HClO), inset: photographs of I) G/CB[8] and II) G/CB[8] /HClO solution under 365 nm light. (c) Fluorescence spectra of G/CB[8] with HClO (80.0 eq.) at different temperatures. (d) Fluorescence spectra of G/CB[8] with HClO (80.0 eq.) in the presence of competitive guest AD (0‐10.0 eq.) (λ_ex_ = 450 nm, [G] = 10 µM, [CB[8]] = 20 µm).

Next, the allosterically controlled luminescence properties of phenyl‐bridged phenothiazine SOF were further investigated by fluorescence experiments. Figure 3a shows that the free cationic guest gives a negligible fluorescence signal at 720 nm. Under confinement within CB[8], the emission peak gradually redshifts to 815 nm, accompanied by enhanced fluorescence intensity. This behavior arises from the large cavity of CB[8], which serves as a supramolecular cross‐linker with the guest, forming a periodic supramolecular structure featuring intermolecular *J*‐type aggregates, leading to a red shift and enhancement of the guest emission. Since the cationic phenothiazine unit in SOF contains a sulfur atom with a variable valence state, it is suitable to investigate the effect of oxidation‐driven guest isomerization on the optical properties of SOF. In the presence of the oxidant HClO (Figure 3b), the new emission peak of SOF at 610 nm was gradually increased. After adding 80.0 eq. of oxidant, the emission peak reaches the maximum, and the solution changes from negligible fluorescence to strong yellow luminescence (Figure 3b, inset). Compared to the original G/CB[8], the oxidation‐driven SOF shows a 205 nm blue‐shift, and the quantum yield increases 46‐fold to 22.2% (Figures S10 and S11), yielding a bright fluorescence periodic supramolecular assembly. The fluorescence lifetime measurements show that after oxidative allostery, the fluorescence lifetime of SOF increased from 0.78 to 11.26 ns at 815 nm (Figure S12a,b), and the newly emerged signal at 610 nm was measured to be 9.93 ns (Figure S12c), manifesting the excited‐state behaviors of SOF could be regulated by the oxidative allostery. The UV–vis spectroscopy experiments show that the absorption peak of G/CB[8] blue shifted from 575 to 450 nm in the presence of HClO (Figure S13), attributing to the oxidation of phenothiazine moiety to the sulfoxide species [33, 34]. Additionally, the original weak fluorescence of G gave a strong fluorescence peak at 555 nm in the presence of HClO (Figure S14), further confirming the tetracation vinylphenylpyridinium‐modified aromatic‐bridged phenothiazine could act as the fluorescence allosteric factor of the SOF. Furthermore, the influence of oxidation‐driven allostery on the assembly behavior of SOF was also explored. In the TEM image (Figure S15), the oxidized G/CB[8] still exhibits a nanosheet morphology. The lamellar thickness measured to be 1.76 nm in AFM experiments (Figure S16) corresponds to the outer diameter of CB[8], indicating that the strong fluorescent assembly remains a monolayer network supramolecular structure. The DLS and Zeta potentials decreased from 513 nm and +13.8 mV to 507 nm and +8.9 mV (Figures S17 and S18), respectively, due to oxidation‐induced changes in the assembly behavior of SOF.

In view of the dynamic reversibility of CB[8]’s host‐guest binding [35, 36], the thermal reversibility and host‐guest regulation responsiveness of the luminescence properties of SOF were further explored. As the temperature increases, the fluorescence intensity of the SOF at 610 nm gradually decreases, reaching a quenching efficiency of 41.6% at 75°C (Figure 3c). When the system is gradually cooled to 25°C, the fluorescence emission of the SOF is partially restored, demonstrating its thermal reversible responsiveness (Figure S19). This reversibility arises because heating induces the disassembly of the oxidized G/CB[8], which is unfavorable for fluorescence, while cooling allows the reformation of the SOF and thereby recovers the luminescence. Addition of the cationic adamantane AD to the SOF caused a gradual decrease in fluorescence and giving a quenching efficiency of 84.7% (Figure 3d). Due to its strong binding constant with CB[8] [37], amantadine hydrochloride (AD) acts as a competitive guest, displacing the luminophore from the confined microenvironment of CB[8] and consequently weakening the system's luminescence. Therefore, by integrating the oxidation‐driven guest allostery and the dynamic host‐guest bonding behavior, the ultra‐bright SOF can be achieved, then realizing the thermally reversible and the host‐guest allosteric dissociation regulating supramolecules luminescence performance.

**FIGURE 4:**
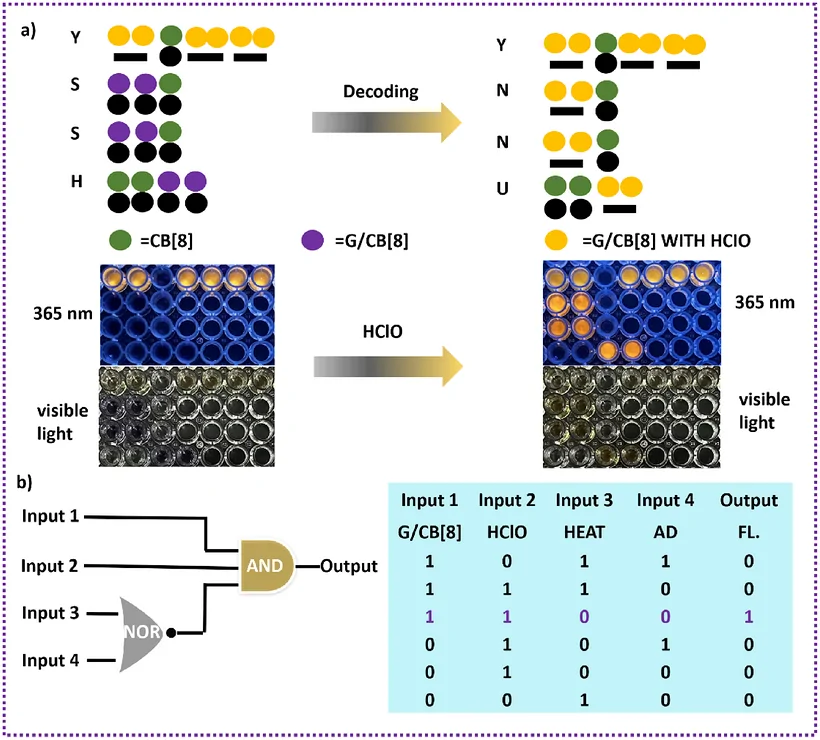
(a) Schematic representation for information encryption: decoding Morse code under 365 nm light, then outputting true information upon addition of the HClO. (b) Heat and completive guest AD regulated “NOR” logic gate.

The luminescent properties of phenylphenothiazine SOF exhibit multi‐stimuli responsiveness. We explored its application in information encryption and complex logic gates. In Figure 4a, CB[8], G/CB[8], and oxidized G/CB[8] were added to the 96‐well plate respectively. Under 365 nm light, the fluorescence of the solution allowed decoding of the first‐level encrypted Moss code as “YSSH”. After the addition of HClO, G/CB[8] emits yellow fluorescence, and then decodes to give the true information, “YNNU”. On the other hand, G/CB[8], HClO, heating, and competitive guest AD are defined as “input 1–4” (Figure 4b). Fluorescence intensity of the SOF at 610 nm is defined as “output”. When the fluorescence signal intensity is higher than “2800”, the “output” is recorded as “1,” and below “2800” is recorded as “1.” Input G/CB[8] and HClO at the same time, the system output is “1.” In the presence of “input 4” or “input 3”, the fluorescence intensity of SOF decreases, and the system output is “0.” Thus, the “heating” and “AD” act as NOR logic gates based on the truth table. Therefore, by taking advantage of the SOF's multi‐stimuli fluorescence responsiveness, it can be applied to information encryption and complex logic gate systems.

## 3 Conclusion

In conclusion, we have successfully constructed a supramolecular controllable allosteric noncovalent framework composed of the tetracationic phenyl‐bridged phenothiazine and macrocyclic host CB[8]. Benefiting from the strong confinement of host, the guest and CB[8] are non‐covalently polymerized at a stoichiometric ratio of 1:2 to form a NIR luminescent network‐like SOF with a large Stokes shift up to 240 nm. Driven by oxidation, phenothiazine is converted to sulfone phenothiazine, switching the SOF from weak NIR luminescence (820 nm) to bright yellow emission at 610 nm and boosting the quantum yield to 22.2%. The luminescent properties of this periodic supramolecular polymer not only exhibit thermos‐reversible allosteric responsiveness but also can be regulated by guest‐allosteric dissociation by introducing cationic adamantane as the competitive guest. Finally, the supramolecular allosteric controllable phenothiazine SOF was successfully applied to information encryption and logic gate systems, providing a new path for the development of intelligent supramolecular materials.

## Author Contributions

**Xinping Lu**: investigation, formal analysis, methodology, visualization. **Jianchong Chen**: investigation, formal analysis. **Jie Yu**: conceptualization, investigation, funding acquisition, writing – review and editing, writing – original draft, supervision, project administration, visualization.

## Conflicts of Interest

The author declares no conflicts of interest.

## Supporting information




**Supporting File**: advs77843‐sup‐0001‐SuppMat.docx.

## Data Availability

The data that support the findings of this study are available from the corresponding author upon reasonable request.
