# Supplementary material for: Supramolecular Allostery Achieves Tunable Luminescence Aromatic Bridged Phenothiazine Noncovalent Frameworks
Source: Adv Sci (Weinh). 2026 Sep 16:e77843. Online ahead of print. doi: 10.1002/advs.77843 (PMC13583087; doi:10.1002/advs.77843)
Supplement: Supplementary file 1 [file ADVS-9999-e77843-s001.docx]

**Supporting Information**

**Supramolecular Allostery Achieves Tunable Luminescence Aromatic Bridged Phenothiazine Noncovalent Frameworks**

Xinping Lu, Jianchong Chen, and Jie Yu*

Yunnan Key Laboratory of Modern Separation Analysis and Substance Transformation. College of Chemistry and Chemical Engineering, Yunnan Normal University, Kunming 650500, P. R. China.

E-mail: yujie5977@126.com

**Materials and Measurements**

The solvents and chemicals were commercially available. The thin layer chromatography was used to judge the chemical reaction. The silica gel (200-300 mesh, Qingdao Haiyang Chemical Co., Ltd) was applied to column chromatography. The ^1^H NMR and ^13^C NMR spectrums were collected from Bruker Avance spectrometers at room temperature. The UV-vis data was performed on the Shimadzu UV-3600 spectrophotometer. The DLS data was collected from the light scattering spectrometer (BI-200SM). The fluorescence spectra and QY were collected from FS5 fluorescence spectrometer and HITACHI fluorescence spectrophotometer (F-4600S). Transmission electron microscope (TEM) pictures were collected from the Philips Tecnai G2 20 S-TWIN microscope. The AFM images were performed on Bruker Dimension Ion AMF.

1. The synthetic route of guest G

**Scheme S1:** the synthetic route of guest G

**Calculation of the binding constant (*K*)**

In the UV-vis titration experiments, the binding constant (*K*) for a stoichiometric 1:2 complex G/CB[8]_2_ (G:CB[8] = 1:2) of G with CB[8] was calculated by using the non-linear least-squares fit of the titration data according to the following formula with the Origin program

Δ*A_obs_* is the UV-vis absorption change of G upon the addition of CB[8]. *K_1_* and *K_2_* are the first-order and the second-order binding constants, respectively. ε_ΔGH_ is the molar absorption coefficient change between the G/CB[8] complex and G. ε_ΔGH2_ is the molar absorption coefficient change between the G/CB[8]_2_ complex and G. [G]_0_ is the initial concentration of guest. [H] is the concentration of the free host.

**1.1 The Synthesis of compound 2**

4-vinylpyridine (0.53 g, 5.1 mmol) and compound 1 (0.50 g, 0.63 mmol) were dissolved in DMF, then tri-o-tolylphosphine (78 mg, 0.25 mmol), the palladium(II)acetate (57 mg, 0.25 mmol), and TEA (3 mL) were added, then the solution was refluxed for 3 days under N_2_. The solution was added to the water, and extracted by dichloromethane. The organic layer was further treated with water and sodium chloride solution, then evaporated, and purified by column chromatography to give compound 2 (0.21 g). ^1^H NMR (400 MHz, DMSO-*d_6_*) δ 8.53 (d, *J* = 5.6 Hz, 8H), 7.77 (s, 4H), 7.51 (d, *J* = 2.2 Hz, 12H), 7.43 (d, *J* = 15.9 Hz, 4H), 7.31 (d, *J* = 8.3 Hz, 4H), 7.15 (d, J = 16.3 Hz, 4H), 6.40 (d, *J* = 8.6 Hz, 4H). HR-MS (MALDI-TOF): m/z calcd for C_58_H_40_N_6_S_2_: 443.1451 ([M+2H]^2+^), found: 443.1455.

**Figure S1**. ^1^H NMR (400 MHz) spectrum of 2 in DMSO-*d_6_* at room temperature.

**Figure S2**. HR-MS of 2.

**1.2 The Synthesis of G**

Compound 2 (150 mg) were dissolved in dry DMF, then CH_3_I (0.5 mL) was injected to the solution and heated at 60 ℃ for 12 h. The solution was poured in diethyl ether to give dark red solid. The saturated NH_4_PF_6_ solution (0.5 mL) was dropped in the iodized salt of G in DMSO, then stirred for 1 h to give precipitate, the filtrate was filtered off to obtain the solid as the hexafluorophosphate of G. The solution of tetrabutylammonium bromide was added in the acetonitrile solution of hexafluorophosphate derivative of G to give precipitate. The reaction mixture was further filtered, dried to give dark red solid G (110 mg) ^1^H NMR (400 MHz, DMSO-*d_6_*) δ 8.83 (d, *J* = 6.5 Hz, 8H), 8.15 (d, *J* = 6.5 Hz, 8H), 7.89 (d, *J* = 15.4 Hz, 8H), 7.62 (s, 4H), 7.44-7.36 (m, 8H), 6.43 (d, J = 8.5 Hz, 4H), 4.25 (s, 12H). ^13^C NMR (100 MHz, DMSO-*d_6_*) δ 152.91, 145.44, 144.15, 140.19, 139.47, 133.92, 131.03, 129.33, 125.91, 123.68, 122.33, 119.86, 117.22, 47.30. HR-MS (MALDI-TOF): m/z calcd for C_62_H_52_N_6_S_2_: 236.0918 (M^4+^), found: 236.0922.

**Figure S3**. ^1^H NMR (400 MHz) spectrum of G in DMSO-*d_6_* at room temperature.

**Figure S4**. ^13^C NMR (100 MHz) spectrum of G in DMSO-*d_6_* at room temperature.

**Figure S5**. HR-MS of G.

**Figure S6**. The Job plot of G/CB[8] ([[G]+[CB[8]]] =20 μM).

**Figure S7**. 2D NOESY spectra of G/CB[8] ([G] = 0.2 mM, [CB[8]] = 0.4 mM) in D_2_O.

**Figure S8**. The Zeta potential of G and G/CB[8] = 1/2 in H_2_O ([G] =10 μM).

**Figure S9**. The DLS of G and G/CB[8] = 1/2 in H_2_O ([G] =10 μM).

**Figure S10**. The QY of G/CB[8] = 1/2 ([G] =10 μM) in H_2_O.

**Figure S11**. The QY of G/CB[8]/HClO ([G] =10 mΜ, [CB[8]] =20 mΜ) in H_2_O.

**Figure S12**. The fluorescence lifetime of a) G/CB[8] and b) G/CB[8]/HClO at 815 nm. The fluorescence lifetime of c) G/CB[8]/HClO at 610 nm ([G] =10 mΜ, [CB[8]] =20 mΜ, λ_ex_ = 450 nm) in H_2_O.

**Figure S13**. The UV-vis spectra of G/CB[8] = 1/2 with the addition of HClO (0-70.0 eq) ([G] =10 μM) in H_2_O.

**Figure S14**. The fluorescence spectra of G with the addition of HClO (0-50.0 eq) ([G] =10 mΜ, λ_ex_ = 425 nm) in H_2_O.

**Figure S15**. The TEM image of G/CB[8] in the presence of HClO.

**Figure S16**. The AFM image of G/CB[8] in the presence of HClO.

**Figure S17**. The DLS of G/CB[8] = 1/2 treated with HClO.

**Figure S18**. The Zeta potential of G/CB[8] = 1/2 treated with HClO.

**Figure S19**. The fluorescence spectra of G/CB[8]/HClO cooling from 75 ℃ to 25 ℃([G] = 10 μM, [CB[8]] = 20 μM).
